# Supplementary material for: Thalamocortical excitability modulation guides human perception under uncertainty
Source: Nat Commun. 2021 Apr 23;12:2430. doi: 10.1038/s41467-021-22511-7 (PMC8065126; doi:10.1038/s41467-021-22511-7)
Supplement: Supplementary file 1 — Supplementary Information [file 41467_2021_22511_MOESM1_ESM.pdf]

Supplementary Information for

Thalamocortical excitability modulation guides human perception under uncertainty

Julian Q. Kosciessa*, Ulman Lindenberger, Douglas D. Garrett*

* Email: kosciessa@mpib-berlin.mpg.de; garrett@mpib-berlin.mpg.de

This PDF file includes:

Supplementary Figures 1 to 5
Supplementary Text 1 to 9
Supplementary Table 1
Supplementary References

Supplementary Text 1. Parameter interrelations. To better understand individual differences in behavioral performance, we explored inter-individual associations between model parameter estimates and 'raw' median RT and mean accuracy. Linear drift rate decreases were inter-individually associated with decreases in accuracy (EEG: r = .35, p = .015, MRI: r = .46, p = .001), but not RT increases (both p > .05), whereas non-decision-time (NDT) increases tracked individual RT increases (EEG: r = .56, p = 3e-5, MRI: r = .64, p = 2e-6), but not accuracy decreases (both p > .05). For single targets, faster RTs were associated with larger drift rates (EEG: r = -.63, p = 3e-6, MRI: r = -.47, p = .002), lower non-decision times (EEG: r = .41, p = .005, MRI: r = .58, p = 3e-5), and lower boundary separation (EEG: r = .58, p = 3e-5, MRI: r = .5, p = 6e-4). More accurate performance for single targets was related to higher drift rates (EEG: r = .74, p = 3e-9; MRI: r = .79, p = 3e-10), but unrelated to boundary separation (EEG: r = .23, p = .121, MRI: r = .18, p = .244) or non-decision times (EEG: r = -.27, p = .069, MRI: r = -.38, p = .011). Amongst model parameters, we observed no parameter relations for single targets (all p > .05). However, we observed intercept-change correlations: participants with larger drift rates for single targets exhibited strong linear drift rate reductions (EEG: r = -.93, p = 4e-22, MRI: r = -.88, p = 1e-15). Moreover, participants with larger boundary separation showed stronger linear increases in non-decision time (r = .46, p = 9e-4, MRI: r = .59, p =2e-5). Non-decision time under selective attention, putatively dominantly reflecting visual encoding time, did not relate to changes in drift rate or NDT (both p > .05). Similarly, boundary separation did not relate to drift rate decreases (both p > .05) and drift rates under selective attention were unrelated to NDT increases (both p > .05).

Supplementary Text 2. Decoding motor preparation signals. We performed a decoding analysis to further explore the emergence of response-specific information across the trial. In particular, we trained a decoder based jointly on the spatial topography of the single-trial broadband time series (~ single-trial CPP), and of the average 8-15 Hz power (estimated using 7 cycle wavelets), both response-locked. Broadband and mu-beta signals were spatially concatenated at each time point, following z-scoring across channels within each measure. This response-locked motor execution classifier was then tested on each time point of signals aligned with stimulus onset (and thus probe onset by virtue of the fixed 3s stimulus presentation period) to assess the ability to classify motor preparation prior to probe onset. For classification analyses, we used linear support-vector machines (SVM) [1] via the libsvm implementation (www.csie.ntu.edu.tw/~cjlin/libsvm). In particular, we decoded ipsi- vs. contralateral response execution in each trial (in line with the lateralized mu-beta analyses). The analysis was separately performed for four splits of trials: (1) all trials, (2) single-target condition trials, (3) multi-target condition trials, in which all cued features converge on the same left or right response for their respective prevalent options during the trial ("response convergence"), (4) multi-target condition trials, in which at least one response would not converge with all other responses. Within each group, the minimum number of available trials across the left and right conditions were randomly selected and split into training (90% of trials) and test sets 100 times for cross-validation. [This approach maximizes the available trials for each split, but includes unequal numbers of trials between splits. Hence, differences in decoding levels between splits should be interpreted with caution.] Results showed that classification accuracy for left vs. right responses was notably above chance and maximal at response, highlighting the adequacy of the response-aligned classifier (Supplementary Figure 2c). Applying this "decoder" to signals aligned to stimulus onset indicated (a) slightly above chance classification for single targets, and (b) slightly below chance classification for multi-targets without response convergence just prior to response (Supplementary Figure 2d). The latter may indicate a representation of the unobserved, alternate motor response, potentially suggesting "changes of mind" following probe presentation. These results jointly allude to the detectable presence of some decision information just prior to probe presentation (but not during a protracted period during stimulus presentation), while also highlighting that much of the eventual response information emerges following probe presentation.

Supplementary Text 3. Behavioral benefits due to convergent responses. To reduce response mapping demands following probe presentation, we fixed response mapping for the two options of each

feature throughout the experiment. Notably, the correct choices (i.e., prevalent options) for multiple cued features can converge on the same left or right response in a given trial ("response convergence"). This potential to prepare motor action prior to probe presentation co-varies as a function of load. To assess the influence of this response convergence on our results, we ran an additional HDDM that simultaneously modelled both a main effect of load, as well as categorical response convergence. Notably, the obtained target load effects on drift rate and NDT were virtually identical to those observed in the selected model in both sessions (reliability of all linear effects: $r >= .9$ $p <.001$), while linear decreases in drift and increases in NDT were also observed as a function of response divergence (i.e., lower drift and higher NDT if the probed attribute required a differential response than the other cued attributes; shown in Supplementary Figure 1e for the EEG session; qualitatively similar results were obtained for MRI session; all linear effects $p < .001$). This suggests that response convergence systematically impacted decision processes, but cannot account for the main effects of target load. However, the large amount of added model parameters introduced partial convergence issues. We therefore chose the simpler model without response convergence for our main analyses.

Supplementary Text 4. NDT increases indicate extended motor preparation demands. We observed a parametric increase in non-decision time (NDT) with target uncertainty (Figure 2b bottom) that described shifts in RT distribution onset (Supplementary Figure 3a). NDT is thought to characterize the duration of processes preceding and following evidence accumulation, i.e., probe encoding and planning/execution of the motor response. We therefore examined sensory probe- and response-related ERP components regarding their modulation by prior target uncertainty. We time-locked the CPP to the NDT group estimate for a single target – for which no button remapping was required – and (2) to the condition-wise NDT estimate. However, we observed no shift in CPP onset (Supplementary Figure 3b), suggesting constant visual encoding time. To probe increases during response preparation, we assessed parametric changes in ERP amplitudes during the interval spanning the final 100 ms prior to response. This interval covered the timeframe of indicated NDT increases, after accounting for the constant probe encoding duration (Supplementary Figure 3b). Notably, we observed a late frontal potential that increased in amplitude (Supplementary Figure 3d) and whose onset corresponded to the temporal NDT shift (Supplementary Figure 3c) after controlling for constant encoding duration (Supplementary Figure 3b). This suggests that baseline NDT estimates approximate the duration of probe encoding [2], whereas NDT increases characterize increased demands for transforming the sensory decision into a motor command [3]. This further suggests that drift diffusion modelling successfully dissociated contributions from evidence integration, sensory encoding, and motor preparation. Interestingly, evidence accumulation consistently peaked at/near response execution, suggesting that additional motor demands may unravel in parallel, rather than succeed finished integration (as is often assumed in sequential sampling models).

Supplementary Text 5. Behavioral PLS of spectral power during stimulus presentation. Task PLS describes the multivariate co-variation of neural indices (here spectral power) with categorical condition labels (here target load in the main analysis). However, neural changes that optimally relate to the main effect of target load may not also optimally capture inter-individual behavioral differences. For example, behavioral relations could be more specific to individual frequency bands than the effect of target load that was jointly observed across frequency ranges. The latter can be probed by means of a behavioral PLS model, which targets the optimal relation of inter-individual behavioral differences to multivariate power changes across frequency bands. To probe whether inter-individual relations of power modulation to behavior would vary from the mean changes as identified via task PLS, we calculated a behavioral PLS by considering the individual linear change in spectral power with target uncertainty. This revealed a similar multivariate loading pattern as observed for the task PLS (Supplementary Figure 4b), with high agreement between individual brainscores ($r = .7$, $p < .001$), suggesting that the identified frequency ranges jointly contributed to behavioral relations.

Supplementary Text 6. Pre-stimulus alpha power increases with load, but does not relate to behavioral changes or power changes during sensation. Furthermore, decreases in pre-stimulus alpha power have been linked to increases in cortical excitability at stimulus onset [4,5]. To probe whether expected uncertainty modulated pre-stimulus alpha power, we performed another task PLS, covering the final second of the fixation interval prior to stimulus onset. This analysis indicated that pre-stimulus alpha power increased alongside uncertainty (Supplementary Figure 4c). Notably, in contrast to current results, elevated levels of anticipatory alpha power are often associated with decreased gamma power upon stimulus onset. Notably, linear models did not indicate associations between pre-stimulus alpha power increases across load with either drift rate decreases [r(137) = 0.02, 95%CI [-0.15, 0.18], p = 0.86], non-decision time increases [r(137) = 0.06, 95%CI [-0.1, 0.23], p = 0.45] or increases on the SPMC [r(137) = -0.13, 95%CI [-0.29, 0.04], p = 0.13]. These results are in line with increasing evidence suggesting that anticipatory alpha power modulation more closely tracks subjective confidence in upcoming decisions than sensory fidelity [6,7].

Supplementary Text 7. Steady-state visually evoked potential (SSVEP) magnitude is not modulated during sensation. Moreover, SSVEP magnitude has been suggested as a signature of encoded sensory information [8], that is enhanced by attention [9,10] and indicates fluctuations in excitability [11]. However, despite a clear SSVEP signature, we did not observe significant effects of encoding demands on the global SSVEP magnitude (Supplementary Figure 4d). As attentional effects on SSVEP magnitude have been shown to vary by SSVEP frequency [12], the 30 Hz range may have been suboptimal here. Furthermore, the SSVEP frequency was shared across different features, thus not allowing us to assess whether uncertainty modulated the selective processing of single features. Implementing feature-specific flicker frequencies may overcome such limitations in future work, and allow to assess the changes in feature-specific processing under uncertainty.

Supplementary Text 8. Rhythm-specific indices in theta and alpha band relate to multivariate spectral power modulation. Finally, as spectral power conflates rhythmic and arrhythmic signal contributions in magnitude, space and time [13], we performed single-trial rhythm detection, observing similar decreases in the duration and power of alpha rhythms (see Supplementary Figure 4e) that were jointly related to stronger increases on the latent factor [duration: r(137) = -0.61, 95%CI [-0.71, -0.49], p = 1.31e-15; power: r(137) = -0.63, 95%CI [-0.72, -0.52], p = 9.66e-17]. Notably, this analysis indicated increases in theta duration, but not power, suggesting that narrowband theta power changes mainly reflected modulations in the duration of non-stationary theta rhythms, rather than changes in their strength. In line with this suggestion, increases on the spectral power factor related to increases in theta duration [r(137) = 0.19, 95%CI [0.02, 0.35], p = 0.03], but not theta SNR [r(137) = 0.09, 95%CI [-0.08, 0.25], p = 0.31].

Supplementary Text 9. A second LV may indicate decreased task engagement due to heightened difficulty at higher uncertainty levels. A 2nd significant LV (p =.012) indicated strong positive loadings in angular gyrus, middle frontal gyrus, and inferior frontal gyrus, as well as occipital cortex (see Supplementary Figure 5b). Negative loadings were observed dominantly in medial PFC, precuneus and V5. This component increased from selective attention to target load 2, but then declined towards higher loads. Decreases in angular gyrus have been strongly to increased visual working memory load [14,15]. Increases in DMN regions, in addition to decreased prefrontal activity suggest that this component reflects relative task disengagement towards high load conditions, while increases in lateral visual cortex may reflect increased entrainment, and lack of top-down inhibition. In line with more negative loadings on this component being detrimental, we observed that inter-individually higher brainscores (i.e., positive loadings) were associated with lower non-decision times during selective attention (r = -0.46, p = .002), while stronger within-subject decreases with load were associated with larger individual NDT increases [r(122) = -0.18, 95%CI [-0.35, -0.01], p = 0.04] but not changes in drift rate [r(122) = 0.01, 95%CI [-0.17, 0.18], p = 0.95]. Larger decreases on this component were moreover related to more constrained

increases in spectral modulation [$r(122) = 0.39$, 95%CI [0.23, 0.53], $p = 6.83e\text{-}6$]. Jointly, this suggests that individual drop-offs in the positive cluster of regions reflects decreased task engagement under increased difficulty, with adverse behavioral consequences.

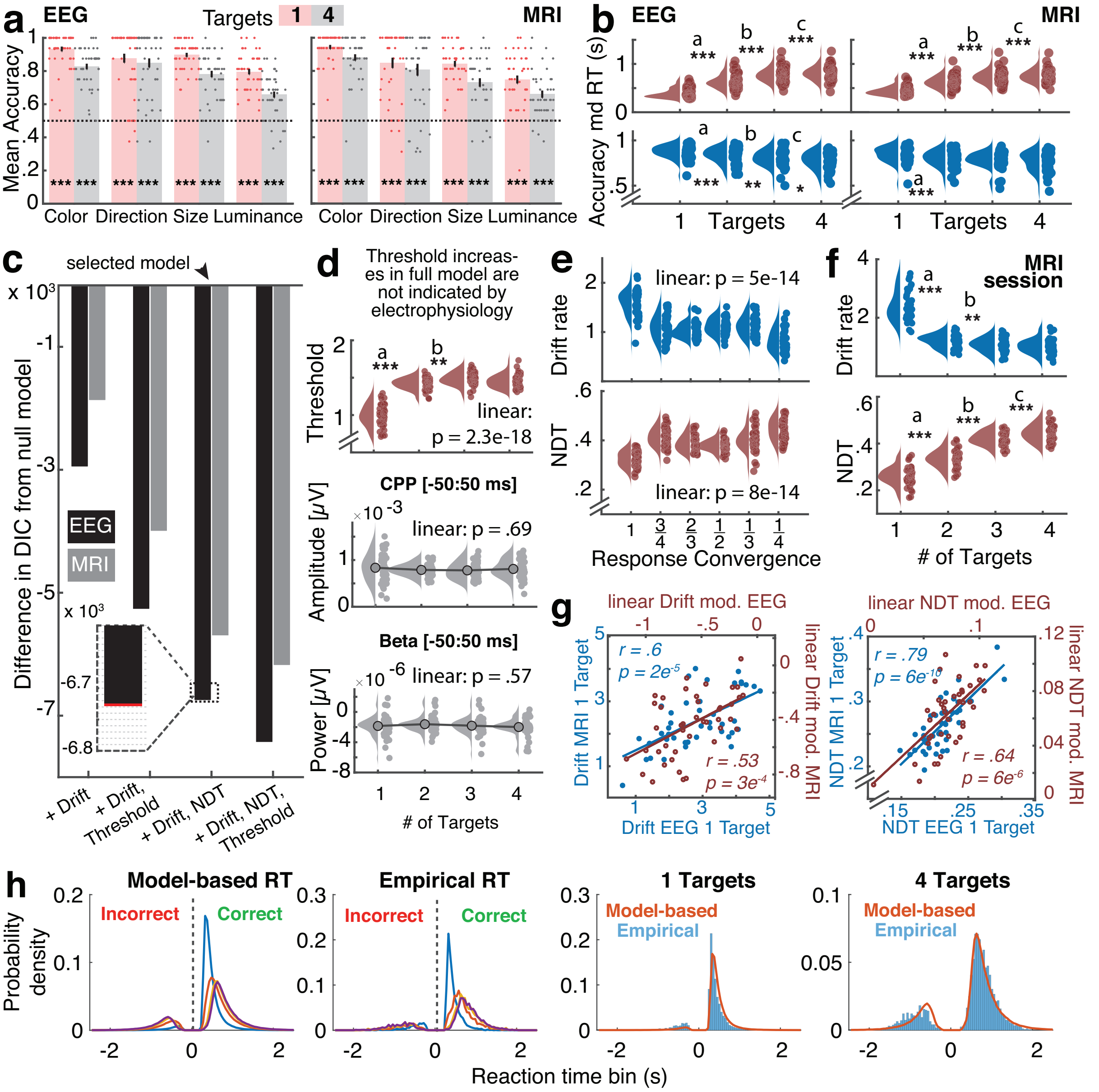

Supplementary Figure 1. Additional behavioral analyses. **(a) Accuracies for single target cue and maximum target uncertainty.** For all features, mean accuracy was above chance accuracy (0.5, indicated by broken lines) at the group level: all *** p < 1e-8 for two-sided paired t-test vs. chance accuracy (Benjamini-Hochberg adjusted). Dots indicate individual accuracies. Bars indicate means +/- SEM. **(b) Median reaction times (mdRT) and mean accuracies by load.** Significant load effects were observed for all variables (all pairwise comparisons Benjamini-Hochberg-adjusted): EEG mdRT (***a: p = 2e-25; ***b: p = 3e-18; ***c: p = .0001; linear: b = 0.14, 95%CI = [0.13, 0.15], t(46) = 26.35, p = 2e-29), MRI mdRT (***a: p = 2e-19; ***b: p = 1e-17; ***c: p = .0002; linear: b = 0.11, 95%CI = [0.1, 0.12], t(43) = 19.57, p = 5e-23), EEG Acc (***a: p = 2e-5; ***b: p = 0.005; ***c: p = .01; linear: b = -0.03, 95%CI = [-0.04, -0.03], t(46) = -9.86, p = 6e-13), MRI Acc (***a: p = 4e-5; linear: b = -0.02, 95%CI = [-0.03, -0.02], t(43) = -7.5, p = 2e-9) **(c-e) HDDM model comparison.** (**c**) DIC-based model comparison indicates that full model, including threshold modulation, provides the best group fit to the behavioral data. However, load-related threshold increases (c) were not supported by EEG-based signatures (see d). The inset shows an additional comparison of the selected model with an alternative model including starting point variation across load levels (displayed in red). Due to very constrained fit improvements, we selected the simpler model without starting point variation for further analyses. (**d**) **Threshold increases in full model are not indicated by electrophysiology.** The full model indicates additional threshold (also called boundary separation) increases with added target load (***a: p = 1.5e-19; ***b: p = .003; linear: b = 0.14, 95%CI = [0.12, 0.17], t(46) = 14.15), with qualitatively identical effects on drift rate and NDT. Boundary separation captures the conservativeness of the decision criterion and has been related to decision conflict during the choice process (e.g., [16]). EEG-based signatures of evidence

integration do not indicate threshold differences. While the full model suggested increased boundary separation, neither of the electrophysiological proxies of evidence bounds mirrors such increases (CPP: linear: b = -8.7e-6, 95%CI = [-5.2e-5, 3.4e-5], t(46) = -0.41; contralateral beta: linear: b = -6.4e-8, 95%CI = [-2.9e-7, 1.6e-7], t(46) = -0.58). While this suggests the absence of threshold increases [17], it alternately questions the sensitivity of electrophysiological threshold estimates, which should be investigated with specific threshold modulations, such as speed-accuracy trade-off instructions, in future work. Data are within-subject centered for visualization (see methods). **(e, f) Differences in response convergence do not account for main effects of target load.** A separate model including both target load and response convergence indicated practically identical NDT and drift rate effects of target amount, while highlighting additional linear effects of response convergence on drift (linear: b = -0.1, 95%CI = [-0.12, -0.08], t(46) = -10.62) and NDT (linear: b = 0.02, 95%CI = [0.01, 0.02], t(46) = 9.79), shown here for the EEG session. Data are within-subject centered for visualization (see methods). **(f-g) Reliability of individual parameter estimates across sessions.** A separate hierarchical DDM was fit to data from each session. (**f**) Similar group-level effects were indicated for the MRI and EEG (cf. Figure 2b) session: whereas drift rate decreased with load (***a: p = 2e-14; ***b: p = .002; linear: b = -0.43, 95%CI = [-0.49, -0.36], t(43) = -12.51, p = 6e-16), non-decision time increased (***a: p = 5e-10; ***b: p = .2e-12; ***c: p = .0004; linear: b = 0.06, 95%CI = [0.06, 0.07], t(43) = 18.37, p = 6e-22). **(g) Session reliability (assessed by Pearson's correlation of inter-individual differences) was high both for single-target performance and for linear changes with target load.** Reliability was also high for threshold estimates (r = .79, 95%CI = [.64, .88], p = 6e-10). 95%CIs: Drift [.63,.77]; Drift mod. [.28,.72]; NDT [.64,.88]; NDT mod. [.41,.79]. **(h) Qualitative model fits in the selected HDDM model (exemplarily shown for the EEG session).** Negative RTs correspond to wrong responses. Model-based ("posterior predictive") values were sampled 50 times within each subject and condition (as implemented in the HDDM package), and probability density (100 RT bins) was estimated first within-subject across all samples, and then averaged across participants. In empirical data, probability densities were estimated across all participants due to the sparse within-subject RT counts. Panels **b, d** and **f** indicate p-values from two-tailed paired *t* tests, pairwise comparisons were Benjamini-Hochberg-adjusted for multiple comparisons. For all EEG data, *n* = 47 participants, for MRI and MRI-EEG data, *n* = 42 participants. Source data are provided as a Source Data file.

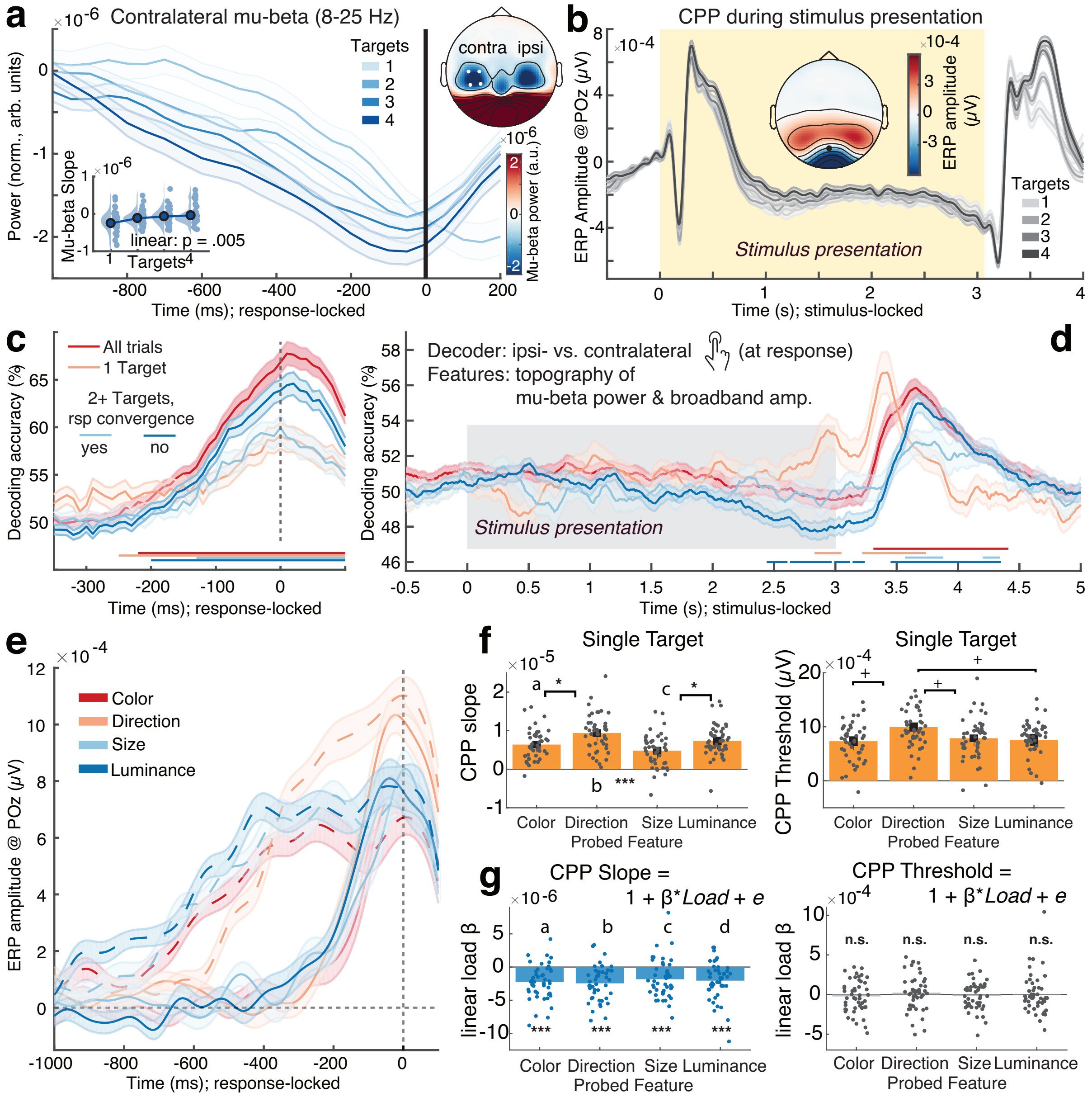

Supplementary Figure 2. Additional drift rate analyses. **(a) The slope of lateralized motor preparation indicates load-related decreases in drift rate.** Slopes of contralateral mu-beta power shallow with increasing attentional load levels. Data are shown as means +/- within-subject SEM. The bottom left inset displays linear slope estimates, estimated via linear regression from -250 ms to -50 ms, relative to response. Data are within-subject centered for visualization (see methods; linear effect: b = 7e-8, 95%CI = [2e-08, 1e-7], t(46) = 2.98, p = .005). The top right inset shows the topography of response-locked mu-beta power, averaged from -50 ms to +50 ms around response. White dots indicate the contralateral channels from which data was extracted. **(b) The centro-parietal positive potential (CPP) does not show clear ramping increases during stimulus presentation.** Data are shown as means +/- within-subject SEM. The yellow background indicated the stimulus presentation period. Note the modulated ramping following the probe onset at the end of stimulus presentation. The inset shows the topography of the grand average ERPs, temporally averaged during the final 2 seconds of the stimulus presentation period. The black dot indicates channel POz, at which the group-wise CPP was maximal (see topography inset in Figure 2c). **(c-d) Motor responses can be decoded briefly prior to probe onset in 1-target condition.** (**c**) Decoding accuracy of executed motor response (left/right) in response-aligned signals from topographies of mu-beta power and broadband amplitudes (see Supplementary Text 2). Maximum decoding accuracy was observed around the time of response for all assessed conditions. (**d**) Decoding performance for stimulus-/probe-aligned signals. Decoders were trained on the response-aligned signals as in **c**. Grey shading indicates the stimulus presentation period, with the offset coinciding with probe onset. Note that due to the discrete periods, pre-probe above/below

chance decoding is unlikely to have resulted exclusively from temporal smoothing due to the wavelet transform. Data are shown as means +/- SEM. For visualization, but not statistics, data in d were smoothed via moving mean averages of 10 samples. Lines below the plot indicate statistically significant deviations from chance decoding (chance level of 50%, two-sided paired t-test, cluster-definition threshold $p < .05$, corrected significance level $p < .05$). (**e-g) Differences between probed stimulus attributes do not account for drift rate decreases under target load.** (**e**) Response-locked CPP as a function of probed attribute, shown for the single target (complete lines) and four target (broken lines) conditions. Data were selected by condition and probed (cf. cued), attribute, ensuring that unique trials contributed to each load condition. Data are shown as means +/- within-subject SEM (centered across target conditions). (**f**) Comparison of CPP slopes and thresholds for different probed features, when the probe target was known in advance. Slopes (and trend-wise thresholds) were increased for direction than for other attribute probes, indicating relatively larger available evidence and more cautious responses (putatively 'easier' feature). The panel indicates p-values from two-tailed paired *t* tests (Slopes: *a: p = .03; ***b: p = .0003; *c: p = .04; Thresholds: all +: p = .05), pairwise comparisons were Benjamini-Hochberg-adjusted for all possible comparisons. Data are within-subject centered for visualization (see methods). (**g**) Load effect of CPP slopes and thresholds for different probed feature attributes. CPP slopes (i.e., evidence drift) exhibited load-related decreases for each probed attribute (***a: p = 7e-7; ***b: p = 2e-7; ***c: p = 6e-5; ***d: p = 8e-6; two-sided paired t-tests vs. zero, Benjamini-Hochberg-adjusted), whereas no threshold modulation was indicated for any of the probed attributes (all p >= .69). *n* = 47 participants for all panels. Source data are provided as a Source Data file.

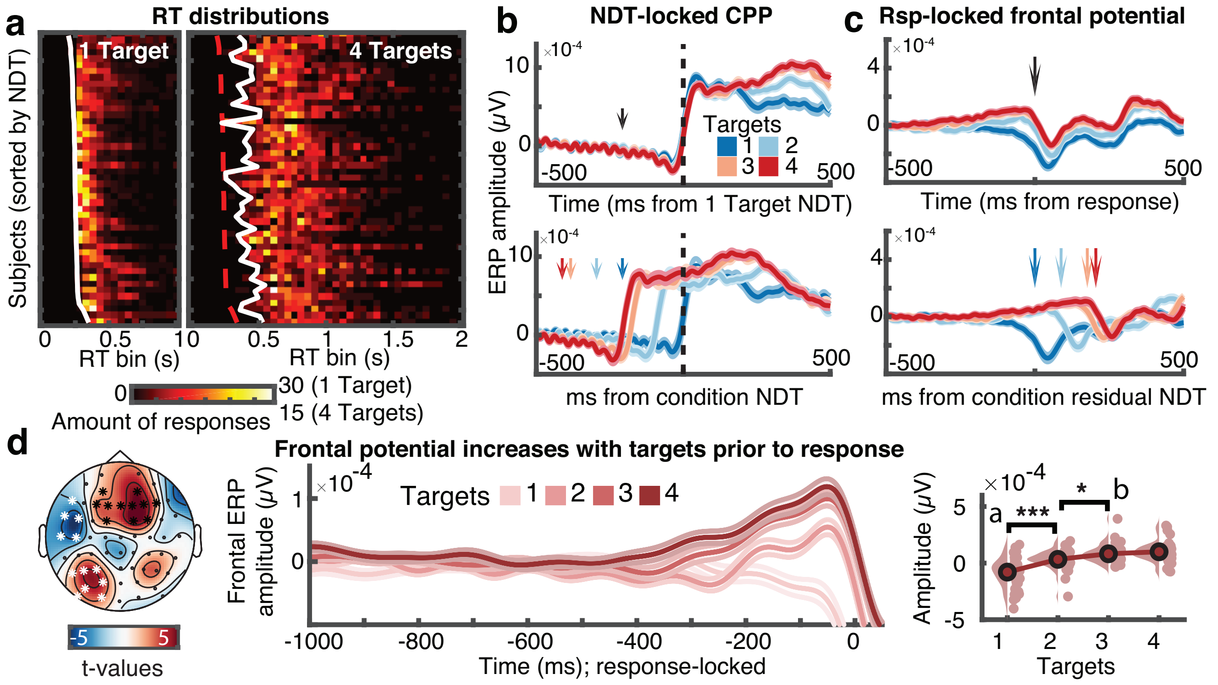

Supplementary Figure 3. Non-decision time (NDT) increases putatively relate to additional motor demands, not temporal delays in CPP onset. **(a) NDT estimates track the onset of individual RT distributions (see also [3]).** Response counts (here shown for the EEG session) were sorted into 40 bins of 50 ms each. White lines indicate individual NDT estimates; the red dotted line indicates NDT estimates for the single-target condition. **(b, c) Relation of CPP (b) and frontal potential (c; depicted in panel d) to indicated NDT increases.** Data are presented as mean values +/- within-subject SEM. Arrows indicate the average probe onset time in b and response time in c, respectively. Black arrows signify averages across conditions, while colored arrows indicate condition-wise averages. **(b)** Average NDT estimates for the single-target condition (b, top), but not condition-specific NDT estimates for multiple targets (b, bottom), track CPP onset latency – a proxy of visual encoding duration. These results suggest that NDT estimates approximate CPP onset only when response preparation can occur in advance (i.e., when only a single target is specified); and that NDT increases do not reflect changes in visual encoding duration. (c) An additional frontal potential may track residual, condition-wise NDT increases above the (presumably constant) encoding duration. The amplitude of the frontal potential "ramps" prior to response when more targets were indicated (c, top). This ramping starts approximately around the time of residual NDT increases (i.e., condition-wise NDT minus single-target NDT; c, bottom). **(d) A frontal potential increase prior to response suggests that observed NDT increases reflect additional motor preparation demands (e.g., button remapping).** Left: Topography of test for linear ERP changes as a function of load during the final 200 ms prior to response. Center: Extracted traces averaged within the frontal cluster shown with black asterisks on the left. Data are presented as mean values +/- within-subject SEM. Right: Post-hoc tests on amplitudes of the frontal potential across the final 100 ms prior to response. Data are within-subject centered for visualization (see methods). The panel indicates p-values from two-tailed paired *t* tests (***a: p = 6e-5; *b: p = .02; linear: b = 6e-5, 95%CI = [4e-5, 8e-5], t(46) = 6.54, p = 5e-8), pairwise comparisons were Benjamini-Hochberg-adjusted. *n* = 47 participants for all panels. Source data are provided as a Source Data file.

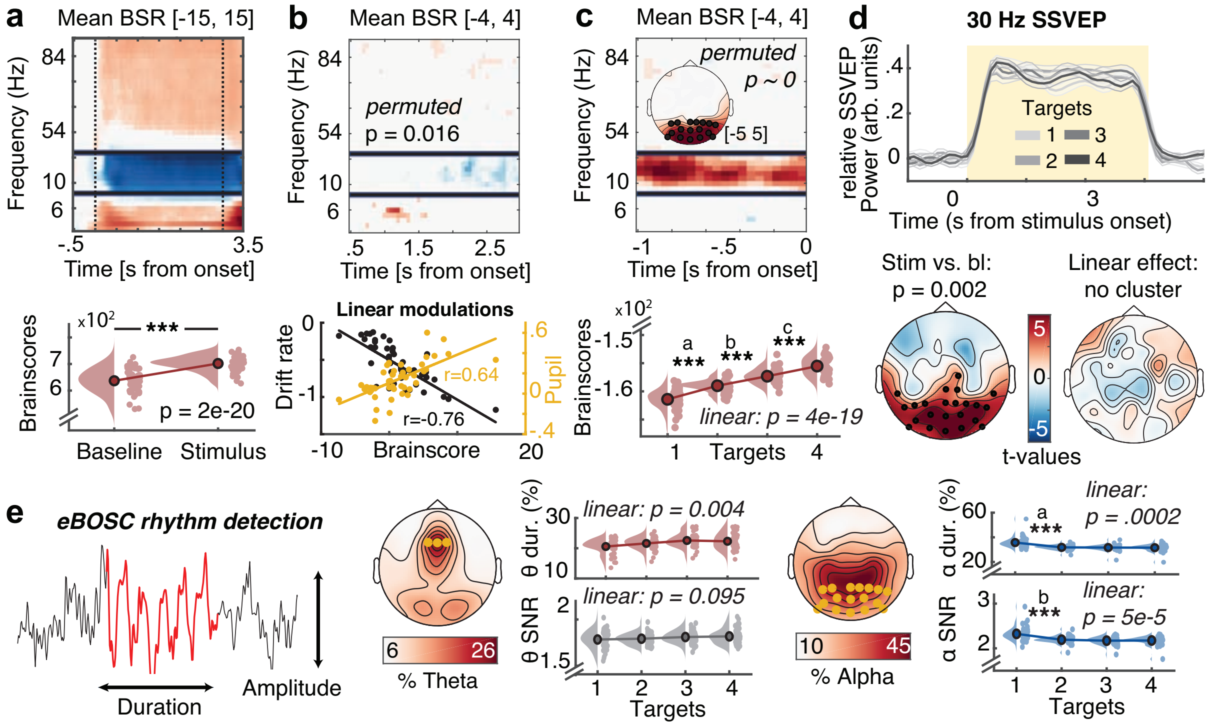

Supplementary Figure 4. Additional spectral power analyses prior and during stimulus presentation. **(a) Task PLS results describing multivariate spectral power changes from the pre-stimulus baseline ('central fixation').** This task PLS model targets optimal multivariate spectral power differences between stimulus presentation and pre-stimulus baseline periods. Upper panel: Bootstrap ratios (BSRs) averaged across the respective channel clusters indicated in Figure 3a. Lower panel: individual brainscore changes as a function of stimulus onset. The model indicates joint theta/gamma power increases (positive loadings in red) and alpha power decreases (negative loadings in blue) following stimulus onset as compared to pre-stimulus baseline. BSR = bootstrap ratio (see methods). **(b) Behavioral PLS results, linking linear multivariate spectral power changes with target # to drift rate decreases and pupil diameter modulation.** This behavioral PLS model targets the optimal statistical relation between multivariate spectral power data (specifically, linear changes as a function of target load) and individual differences in drift rate and pupil modulations. Upper panel: Bootstrap ratios (BSRs) averaged across the respective channel clusters indicated in Figure 3a. Lower panel: Individual brainscore relations (Pearson's correlations) to linear changes in drift rate (95%CI = [-.86, -.6]) and pupil dimeter (95%CI = [.43, .78]). **(c) Parieto-occipital pre-stimulus alpha power increases with target load but is not related to drift changes (see Supplementary Text 4)*.*** The time-frequency plot shows the results of the task PLS relating multivariate pre-stimulus ('fixation') power values to target load categories, indicating major loadings in the alpha band. The inset shows the topography of mean BSR values and the channels across which results were averaged for the time-frequency plot. The lower plot illustrates brainscore increases as a function of target number (***a: p = 7e-9; ***b: p = 7e-7; ***c: p = 4e-6; linear: b = 1.93, 95%CI = [1.67, 2.19], t(46) = 14.84). Data are within-subject centered for visualization (see methods). Linear brainscore increases were not related to drift changes (not shown here, see Supplementary Text 4). **(d) Steady-state visually evoked potential (SSVEP) amplitude is not modulated by attentional load*.*** Top: Time-resolved, spectrally-normalized, SSVEP power, averaged across occipital channels (O1, Oz, O2), indicates clear SSVEP increases specifically during stimulus presentation. Data are presented as mean values +/- within-subject SEM. Bottom left: Topography of stimulus-evoked SSVEP contrast minus baseline. Black dots indicate channels within a significant cluster as indicated by two-sided paired t tests, corrected for multiple comparisons via cluster-based permutations. Bottom right: No linear load-related SSVEP modulation was indicated by CBPA. **(e) Modulation of rhythm-specific duration and power by target number.** Left: Schematic of the assessment of amplitude and duration from non-stationary rhythmic events. Right: Topographies of relative theta (θ) and alpha (α) duration, averaged across target levels. Orange dots indicate the channels used to extract the data in e, which were the same channels also used in Figure 3a/b. Target load decreased alpha duration (***a: p = 6e-7; linear: b = -1.27, 95%CI = [-1.89, -0.64], t(46) = -4.1) and

SNR (***b: $p = 5e-5$; linear: $b = -0.05$, 95%CI = [-0.07, -0.03], $t(46) = -4.46$) and increased theta duration (linear: $b = 0.61$, 95%CI = [0.21, 1.02], $t(46) = 3.07$), but not SNR (linear: $b = 0.01$, 95%CI = [0, 0.03], $t(46) = 1.71$). Data are within-subject centered for visualization (see methods). Panels **a, c** and **e** indicate p-values from two-tailed paired *t* tests, pairwise comparisons were Benjamini-Hochberg-adjusted for multiple comparisons. In panels **b** and **c**, permuted p-values result from comparing the strength of latent variables against random permutations (see methods). *n* = 47 participants for all panels. Source data are provided as a Source Data file.

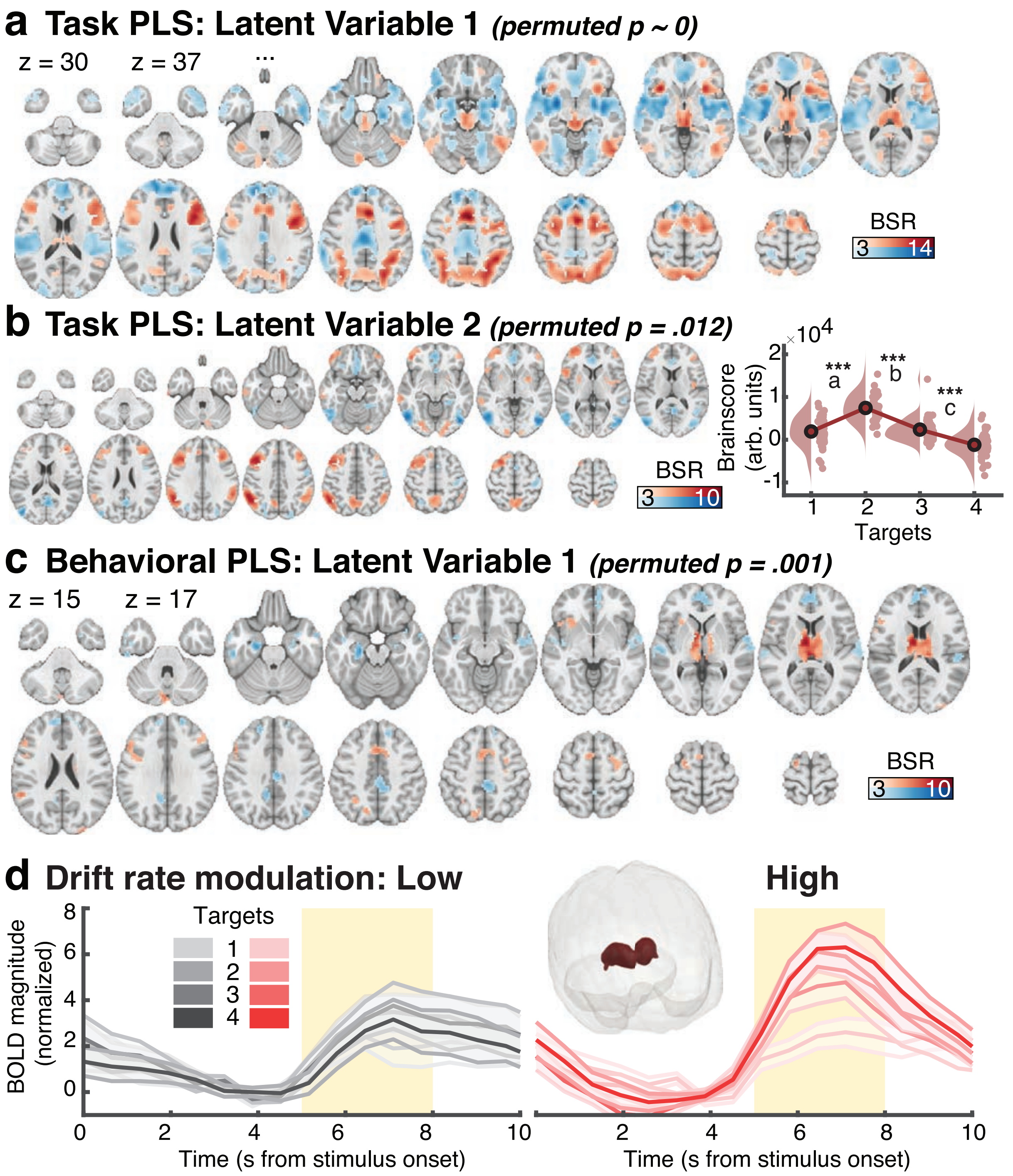

Supplementary Figure 5. Additional BOLD analyses. **(a-c) Full multivariate brainscore loadings for the two significant latent variables (LVs) produced by the task PLS (a, b) and behavioral PLS (c).** Permuted p-values result from comparing the strength of latent variables against random permutations (see methods). (**b right**) The brainscore loadings of the second LV designate an initial increase followed by a subsequent decrease towards higher target loads (n = 42 participants, ***a: p = 9e-8, ***b: p = 2e-9, ***c: p = 4e-6; p-values from two-tailed paired *t* tests, Benjamini-Hochberg-adjusted for multiple comparisons). Data are within-subject centered for visualization (see methods). Thus, the negative components of the pattern expressed on the left of panel **b** become more strongly activated at low and high loads, whereas the positive components are maximally expressed when two targets are relevant. **(d) Thalamic BOLD magnitude for a median split of high- (*n* = 21) and low- (*n* = 21) drift rate modulators.** The visualization is the same as in Figure 7c, showing means +/- SEMs. The inset shows the thalamic ROI in a glass brain view. Source data are provided as a Source Data file.

Supplementary Table 1. PLS model peak activations, bootstrap ratios, and cluster sizes.

			MNI Coordinates				
Model	Region	Hem	X	Y	Z	BSR	#Voxels
taskPLS LV1<br><br>BSR [-3 3]	Mid-cingulate cortex (MCC)	L	-6	15	42	13.42	2708
	Inferior Parietal Lobule (IPS)	L	-45	-45	45	11.14	2664
	Insula Lobe (anterior)<br>[33.0 21.0 -3]	R	33	18	-3	10.86	175
	Inferior Occipital Gyrus<br>[-54 -69 -12]	L	-57	-69	-12	10.1	702
	Thalamus<br>[-8 -27 -2]	L	-6	-30	-3	9.93	1121
	Superior Frontal Gyrus	R	27	-3	54	9.47	880
	Inferior Temporal Gyrus	R	51	-60	-12	6.72	265
	Superior Orbital Gyrus	L	-27	54	-3	6.2	232
	Cerebellum (Crus 1)	R	6	-81	-24	6.12	109
	PCC<br>[-6.0 -35.0 28.0]	L	-9	-33	27	5.72	62
	Cerebellum (VI)	R	30	-63	-30	5.64	59
	Cerebellar Vermis (9)		0	-57	-36	4.32	32
	Cerebellum (Crus 2)	L	-6	-84	-33	3.82	26
	Pallidum<br>[24.0 3.0 -6.0]; bilateral	R	24	0	-9	-11.74	3882
	Insula Lobe	L	-33	-18	6	-11.05	3776
	Superior Frontal Gyrus	L	-12	36	54	-10.6	2096
	MCC	L	0	-15	36	-9.72	706
	Lingual Gyrus	R	21	-84	-6	-7.43	440
	Superior Occipital Gyrus	R	27	-96	15	-5.54	318
	Middle Frontal Gyrus	L	-33	24	39	-5.48	44
	Angular Gyrus	L	-48	-63	27	-5.31	106
	Superior Parietal Lobule	L	-21	-45	63	-5.12	94
	Postcentral Gyrus	R	21	-39	63	-4.98	89
BSR [-6 6] (additional clusters that were merged in +/- 3 threshold)	IFG	L	-45	9	30	12.576	790
	Insula Lobe	L	-33	18	-3	10	93
	IFG	R	42	27	18	7	32
	IFG	R	51	33	-9	-8.69	125
	SMG	R	57	-39	39	-7.80	56
	Inferior Temporal Gyrus	L	-57	-6	-33	-7.57	96
taskPLS LV2	Angular Gyrus	R	54	-51	36	8.69	638
	Middle Frontal Gyrus	R	39	18	39	8.24	1238
	IFG (p. Orbitalis)	R	42	45	-12	6.37	141
	SupraMarginal Gyrus	L	-60	-45	33	6.36	317
	Middle Frontal Gyrus	L	-42	24	33	6.21	477
	Inferior Occipital Gyrus	L	-27	-90	-12	5.66	110
	Precuneus	R	3	-60	45	5.54	383
	Middle Temporal Gyrus	R	60	-33	-12	5.26	154
	IFG (p. Triangularis)	R	48	18	3	5.07	115
	Lingual Gyrus	R	21	-84	-6	4.99	77
	Putamen	L	-30	3	-3	4.62	115
	Cerebelum (Crus 2)	L	-9	-81	-27	4.22	34
	Putamen	R	24	0	6	3.93	30
	Inferior Occipital Gyrus	L	-48	-75	-6	-7.92	378
	Inferior Occipital Gyrus	R	51	-72	-15	-7.61	706
	Olfactory cortex	L	-3	18	-12	-5.63	502
	Precuneus	L	-6	-63	21	-5.56	220
	Superior Parietal Lobule	R	27	-54	63	-4.46	39
	Fusiform Gyrus	L	-24	-45	-15	-4.43	83
	Postcentral Gyrus	L	-57	-3	42	-4.38	58
	Postcentral Gyrus	L	-45	-27	57	-4.36	85
	Superior Orbital Gyrus	R	21	27	-15	-4.32	25
	Superior Occipital Gyrus	R	27	-69	36	-4.29	58
	Precentral Gyrus	L	-42	0	30	-4.23	28
	Middle Temporal Gyrus	L	-54	-57	12	-4.18	38
			-69	-42	9	-4.13	51
	Middle Occipital Gyrus	L	-30	-81	36	-4.1	60
	Posterior-Medial Frontal	L	-6	6	60	-3.95	33
	Hippocampus	L	-27	-18	-21	7.04	111

behavioral PLS: LV1	Inferior Temporal Gyrus [-56 -24 -30]	L	-57	-24	-30	5.5	40
	Superior Medial Gyrus	R	3	63	15	5.43	345
	ParaHippocampal Gyrus	R	21	-12	-24	5.35	35
	MCC	R	3	-33	48	5.3	174
	Middle Temporal Gyrus	L	-60	0	-30	4.77	27
	MCC	L	-12	-45	36	4.72	64
	Superior Frontal Gyrus	R	18	51	30	4.68	33
	Fusiform Gyrus	R	24	12	-45	4.67	30
	Middle Temporal Gyrus	R	57	-3	-15	4.64	239
	Superior Frontal Gyrus	L	-21	42	36	4.61	26
	Superior Temporal Gyrus	L	-57	-21	3	4.6	61
	Angular Gyrus	R	39	-72	39	4.59	36
	Middle Temporal Gyrus	L	-51	-3	-21	4.52	72
	Temporal Pole	R	36	6	-21	4.42	25
	Superior Medial Gyrus	L	9	36	45	4.25	29
	Thalamus	L	-9	-9	12	-9.73	591
	Superior Frontal Gyrus	L	-24	-3	69	-5.59	38
	Posterior-Medial Frontal	L	-3	15	45	-5.22	154
	Superior Occipital Gyrus	R	27	-96	21	-5.15	39
	SupraMarginal Gyrus	L	-60	-48	24	-5.13	28
	Cerebelum (Crus 2)	L	-6	-84	-33	-5.09	35
	Superior Parietal Lobule	L	-18	-69	48	-5.07	36
	IFG (p. Opercularis)	L	-57	15	33	-4.87	173
	Insula Lobe	L	-30	21	-3	-4.37	44
	Inferior Parietal Lobule	L	-33	-54	45	-4.03	30
	Superior Frontal Gyrus	R	24	0	54	-3.9	51
	Middle Frontal Gyrus	R	45	36	33	-3.78	35

Note: Locations where peaks had to be shifted for a label are indicated with coordinates in the label.

Supplementary References

1 Muller, K. R., Mika, S., Ratsch, G., Tsuda, K. & Scholkopf, B. An introduction to kernel-based learning algorithms. *IEEE Trans. Neural Networks* **12**, 181-201, doi:Doi 10.1109/72.914517 (2001).

2 Nunez, M. D., Vandekerckhove, J. & Srinivasan, R. How attention influences perceptual decision making: Single-trial EEG correlates of drift-diffusion model parameters. *J. Math. Psychol.* **76**, 117-130, doi:10.1016/j.jmp.2016.03.003 (2017).

3 Lui, K. K. *et al.* Timing of readiness potentials reflect a decision-making process in the human brain. *bioRxiv* (2018).

4 Lange, J., Oostenveld, R. & Fries, P. Reduced occipital alpha power indexes enhanced excitability rather than improved visual perception. *J. Neurosci.* **33**, 3212-3220, doi:10.1523/JNEUROSCI.3755-12.2013 (2013).

5 Iemi, L., Chaumon, M., Crouzet, S. M. & Busch, N. A. Spontaneous Neural Oscillations Bias Perception by Modulating Baseline Excitability. *J. Neurosci.* **37**, 807-819, doi:10.1523/JNEUROSCI.1432-16.2016 (2017).

6 Benwell, C. S. Y. *et al.* Prestimulus EEG Power Predicts Conscious Awareness But Not Objective Visual Performance. *eNeuro* **4**, doi:10.1523/ENEURO.0182-17.2017 (2017).

7 Limbach, K. & Corballis, P. M. Prestimulus alpha power influences response criterion in a detection task. *Psychophysiology* **53**, 1154-1164, doi:10.1111/psyp.12666 (2016).

8 O'Connell, R. G., Dockree, P. M. & Kelly, S. P. A supramodal accumulation-to-bound signal that determines perceptual decisions in humans. *Nat. Neurosci.* **15**, 1729-1735, doi:10.1038/nn.3248 (2012).

9 Morgan, S. T., Hansen, J. C. & Hillyard, S. A. Selective attention to stimulus location modulates the steady-state visual evoked potential. *Proc. Natl. Acad. Sci. U. S. A.* **93**, 4770-4774, doi:10.1073/pnas.93.10.4770 (1996).

10 Muller, M. M. *et al.* Feature-selective attention enhances color signals in early visual areas of the human brain. *Proc. Natl. Acad. Sci. U. S. A.* **103**, 14250-14254, doi:10.1073/pnas.0606668103 (2006).

11 Zhigalov, A., Herring, J. D., Herpers, J., Bergmann, T. O. & Jensen, O. Probing cortical excitability using rapid frequency tagging. *NeuroImage* **195**, 59-66, doi:10.1016/j.neuroimage.2019.03.056 (2019).

12 Ding, J., Sperling, G. & Srinivasan, R. Attentional modulation of SSVEP power depends on the network tagged by the flicker frequency. *Cereb. Cortex* **16**, 1016-1029, doi:10.1093/cercor/bhj044 (2006).

13 Kosciessa, J. Q., Grandy, T. H., Garrett, D. D. & Werkle-Bergner, M. Single-trial characterization of neural rhythms: Potential and challenges. *NeuroImage* **206**, 116331, doi:10.1016/j.neuroimage.2019.116331 (2020).

14 Todd, J. J. & Marois, R. Capacity limit of visual short-term memory in human posterior parietal cortex. *Nature* **428**, 751-754, doi:10.1038/nature02466 (2004).

15 Sheremata, S. L., Somers, D. C. & Shomstein, S. Visual Short-Term Memory Activity in Parietal Lobe Reflects Cognitive Processes beyond Attentional Selection. *J. Neurosci.* **38**, 1511-1519, doi:10.1523/JNEUROSCI.1716-17.2017 (2018).

16 Cavanagh, J. F. *et al.* Subthalamic nucleus stimulation reverses mediofrontal influence over decision threshold. *Nat. Neurosci.* **14**, 1462-1467, doi:10.1038/nn.2925 (2011).

17 McGovern, D. P., Hayes, A., Kelly, S. P. & O'Connell, R. G. Reconciling age-related changes in behavioural and neural indices of human perceptual decision-making. *Nat Hum Behav* **2**, 955-966, doi:10.1038/s41562-018-0465-6 (2018).
