# Supplementary material for: Thalamocortical excitability modulation guides human perception under uncertainty
Source: Nat Commun. 2021 Apr 23;12:2430. doi: 10.1038/s41467-021-22511-7 (PMC8065126; doi:10.1038/s41467-021-22511-7)
Supplement: Supplementary file 3 — Reporting Summary [file 41467_2021_22511_MOESM3_ESM.pdf]

nature research

Corresponding author(s): Julian Kosciessa, Douglas Garrett

Last updated by author(s): Feb 12, 2021

Reporting Summary

Nature Research wishes to improve the reproducibility of the work that we publish. This form provides structure for consistency and transparency in reporting. For further information on Nature Research policies, see our Editorial Policies and the Editorial Policy Checklist.

Statistics

For all statistical analyses, confirm that the following items are present in the figure legend, table legend, main text, or Methods section.

n/a	Confirmed	
☐	☒	The exact sample size (*n*) for each experimental group/condition, given as a discrete number and unit of measurement
☐	☒	A statement on whether measurements were taken from distinct samples or whether the same sample was measured repeatedly
☐	☒	The statistical test(s) used AND whether they are one- or two-sided<br>*Only common tests should be described solely by name; describe more complex techniques in the Methods section.*
☐	☒	A description of all covariates tested
☐	☒	A description of any assumptions or corrections, such as tests of normality and adjustment for multiple comparisons
☐	☒	A full description of the statistical parameters including central tendency (e.g. means) or other basic estimates (e.g. regression coefficient) AND variation (e.g. standard deviation) or associated estimates of uncertainty (e.g. confidence intervals)
☐	☒	For null hypothesis testing, the test statistic (e.g. *F*, *t*, *r*) with confidence intervals, effect sizes, degrees of freedom and *P* value noted<br>*Give P values as exact values whenever suitable.*
☐	☒	For Bayesian analysis, information on the choice of priors and Markov chain Monte Carlo settings
☒	☐	For hierarchical and complex designs, identification of the appropriate level for tests and full reporting of outcomes
☐	☒	Estimates of effect sizes (e.g. Cohen's *d*, Pearson's *r*), indicating how they were calculated

Our web collection on statistics for biologists contains articles on many of the points above.

Software and code

Policy information about availability of computer code

Data collection	BrainVisionRecorder; PsychToolbox 3.0.11; Experiment code is available from https://git.mpib-berlin.mpg.de/LNDG/multi-attribute-task.
Data analysis	MATLAB R2016B, R2017B; FieldTrip 20170904; SPM 12; PLS toolbox v.6.15 (incl. custom adaptations for recent MATLAB versions available upon request); MEG-PLS toolbox [version 2.02b]; eBOSC (v0.9; available from https://github.com/jkosciessa/eBOSC); mMSE (available from https://github.com/LNDG/mMSE); SPM Anatomy Toolbox (Version 2.2c); FSL 5 (RRID:SCR_002823); ANTs 2.1.0; EyeLink v4.40; MEG-PLS v2.02b; R 4.0.3; HDDM v.0.6.0, custom implementation of FASTER. All analysis code is available at https://git.mpib-berlin.mpg.de/LNDG/stateswitch.

For manuscripts utilizing custom algorithms or software that are central to the research but not yet described in published literature, software must be made available to editors and reviewers. We strongly encourage code deposition in a community repository (e.g. GitHub). See the Nature Research guidelines for submitting code & software for further information.

Data

Policy information about availability of data

All manuscripts must include a data availability statement. This statement should provide the following information, where applicable:

- Accession codes, unique identifiers, or web links for publicly available datasets
- A list of figures that have associated raw data
- A description of any restrictions on data availability

Primary EEG, fMRI and behavioral data are available from https://osf.io/ug4b8/ (doi: 10.17605/OSF.IO/UG4B8). Structural MRI data are exempt from public sharing according to informed consent. Source data for select panels in Figures 2-7 and Supplementary Figures 1-5 are provided with this paper. All data are available from the corresponding authors upon reasonable request.

Field-specific reporting

Please select the one below that is the best fit for your research. If you are not sure, read the appropriate sections before making your selection.

☒ Life sciences ☐ Behavioural & social sciences ☐ Ecological, evolutionary & environmental sciences

For a reference copy of the document with all sections, see nature.com/documents/nr-reporting-summary-flat.pdf

Life sciences study design

All studies must disclose on these points even when the disclosure is negative.

Sample size	A sample size of N = 50 was targeted to allow for across-subject correlations with a power of 80% between multi-modal indices given an expected correlation coefficient of around .4 at p = .05 (two-sided). Only N = 47 subjects were collected in the final sample (prior to any analysis being performed).
Data exclusions	No data were excluded from the analyses. N = 5 subjects dropped out following the initial EEG session with no reasons given. Analyses are based on the full available sample for each modality.
Replication	No independent replication was attempted in the present manuscript. A conceptual replication is currently attempted using an independent sample of older adults.
Randomization	No allocation to experimental groups was undertaken at the level of subjects. Across experimental conditions, presentation was pseudo-randomized such that every size and constellation of the cue set was presented across blocks. Within each run of four blocks, every set size was presented once, but never directly following a block of the same set size. In every block, each feature in the active set acted as a probe in at least one trial. Moreover, any attribute equally often served as a probe across all blocks. Winning options for each feature were balanced across trials, such that (correct) button responses were equally distributed across the experiment.
Blinding	No group allocation was performed in this study, thus no blinding was performed

Reporting for specific materials, systems and methods

We require information from authors about some types of materials, experimental systems and methods used in many studies. Here, indicate whether each material, system or method listed is relevant to your study. If you are not sure if a list item applies to your research, read the appropriate section before selecting a response.

Materials & experimental systems

n/a	Involved in the study	
☒	☐	Antibodies
☒	☐	Eukaryotic cell lines
☒	☐	Palaeontology and archaeology
☒	☐	Animals and other organisms
☐	☒	Human research participants
☒	☐	Clinical data
☒	☐	Dual use research of concern

Methods

n/a	Involved in the study	
☒	☐	ChIP-seq
☒	☐	Flow cytometry
☐	☒	MRI-based neuroimaging

Human research participants

Policy information about studies involving human research participants

Population characteristics	47 young adults (18-35 years, mean age = 25.8 years, SD = 4.6, 25 women), describing themselves to be in healthy condition.
Recruitment	Participants were randomly recruited via telephone calls using the participant database at the Max Planck Institute for Human Development. Participants needed to be in good health to fulfill the internal criteria for EEG and fMRI studies. Participant recruitment was performed by a team that did not include the principal researchers, and participants were blind to the specific study hypotheses. We do not perceive a major self-selection bias to participate in the study beyond a general interest in cognitive psychology/neuroscience studies, which is unlikely to have impacted the results.
Ethics oversight	Deutsche Gesellschaft für Psychologie (DGPS)

Note that full information on the approval of the study protocol must also be provided in the manuscript.

Magnetic resonance imaging

Experimental design

Design type	task; event-related
Design specifications	In one MRI session (and an additional EEG session, participants performed 4 runs a 32 blocks containing 8 trials, i.e., a total of 256 trials. Each trial was structured as follows: cue onset during which the relevant targets were centrally presented (1 s), fixation phase (2 s), dynamic stimulus phase (3 s), probe phase (incl. response; 2 s); ITI (un-jittered; 1.5 s). The current analyses concern the dynamic stimulus phase.
Behavioral performance measures	Variables: button press (left, right; recoded as correct/incorrect), reaction time. Mean accuracy was analyzed to establish that subjects performed the task as expected

Acquisition

Imaging type(s)	structural, functional
Field strength	3 Tesla
Sequence & imaging parameters	Whole-brain task fMRI data (4 runs á ~11,5 mins, 1066 volumes per run) were collected via a 3T Siemens TrioTim MRI system (Erlangen, Germany) using a multi-band EPI sequence (factor 4; TR = 645 ms; TE = 30 ms; flip angle 60°; FoV = 222 mm; voxel size 3x3x3 mm; 40 transverse slices. The first 12 volumes (12 × 645 ms = 7.7 sec) were removed to ensure a steady state of tissue magnetization (total remaining volumes = 1054 per run). A T1-weighted structural scan was also acquired (MPRAGE: TR = 2500 ms; TE = 4.77 ms; flip angle 7°; FoV = 256 mm; voxel size 1x1x1 mm; 192 sagittal slices). A T2-weighted structural scan was also acquired (GRAPPA: TR = 3200 ms; TE = 347 ms; FoV = 256 mm; voxel size 1x1x1 mm; 176 sagittal slices)
Area of acquisition	A whole-brain scan was acquired.

Diffusion MRI ☐ Used ☒ Not used

Preprocessing

Preprocessing software	FSL 5 (RRID:SCR_002823): McFLIRT, 7 mm smoothing, .01 Hz high-pass filtering (8th order zero-phase Butterworth filter)
Normalization	We registered individual functional runs to the individual, ANTs brain-extracted T2w images (6 DOF), to T1w images (6 DOF). Finally, those images were normalized to 3mm standard space (ICBM 2009c MNI152 nonlinear symmetric) using nonlinear transformations in ANTs.
Normalization template	ICBM 2009c MNI152 nonlinear symmetric
Noise and artifact removal	Noise and artifacts from non-neural sources were identified based on visual inspection of independent components. Independent Component Analysis (ICA) was performed using FSL-MELODIC and noise components were manually identified via visual inspection. Labeling criteria are described in the manuscript. 6 DOF motion parameters, avg. white matter and CSF signal were regressed from the data. Nuisance regressors were included in the 1st level analysis (24 motion parameters, DVARS estimates).
Volume censoring	DVARS outliers (Afyouni & Nichols, 2018) were censored and interpolated as described in Power et al. (2014) and Parkes et al. (2018)

Statistical modeling & inference

Model type and settings	1st level mass univariate (SPM) model: beta weights of stimulus onset BOLD response for each cue set size (autoregressive modelling: FAST; canonical HRF; 24 motion + DVARS + HRF derivative as nuisance regressors); 2nd level: multivariate relation between 1st level beta weights (see below)
Effect(s) tested	(1) Task PLS: main effect of cue set size; (2) Relation of linear set-size-related changes in 1st level betas to independent variables of interest (behavior, EEG, pupil, see below)

Specify type of analysis: ☐ Whole brain ☒ ROI-based ☐ Both

Anatomical location(s)	Thalamic parcels were selected based on existing parcellation schemes
Statistic type for inference (See Eklund et al. 2016)	Cluster-wise inference at 2nd level; cluster definition: minimum distance of 10 mm; size threshold: 25 voxels; statistical threshold: PLS bootstrap ratio > 3 (=>99.5% threshold)
Correction	permutation correction within PLS toolbox (see McIntosh & Lobaugh, 2004)

Models & analysis

n/a	Involved in the study
☒	☐ Functional and/or effective connectivity
☒	☐ Graph analysis
☐	☒ Multivariate modeling or predictive analysis

Multivariate modeling and predictive analysis

We performed a multivariate partial least squares (PLS) analysis across subjects with the following independent variables: drift-diffusion estimates (drift, threshold, non-decision time; baseline and modulation), spectral power modulation factor (EEG), spectral slope modulation (EEG), sample entropy modulation (EEG), pupil modulation.
